# Report of Two Deaths from Partial Chloroform-Anæsthesia, with Remarks

**Published:** 1890-01

**Authors:** J. T. Harrington

**Affiliations:** El Paso, Texas


For Daniel's Texas Medical Journal.

### REPORT OF TWO DEATHS FROM PARTIAL CHLOROFORM ANÆSTHESIA, WITH REMARKS.

BY J. T. HARRINGTON, M. D., EL PASO, TEXAS.

AUGUST 22, 1889. Mrs. J——, colored, aged 42 years, ovarian tumor. Patient has been confined to her bed most of the time during the past year. Is very much emaciated, and bears every indication of devitalization. After consultation an immediate operation is decided upon as the only means of saving the woman's life. Patient placed in the dorsal decubitus and chloroform anæsthesia commenced with a napkin folded in a paper cone. Pulse rapid and weak, the respiration hurried but no resistance to the chloroform; after a few inhalations the breathing became difficult and stertorous, no pulse and no distinguishable heart-beat. The usual methods of resuscitation were resorted to—lowering the patient's head, elevation of jaw by pressure behind the condyles, hypodermic injections of brandy, artificial respiration, etc., but all to no purpose, for within a minute and a half from the time the patient was placed upon the operating table she was dead. Autopsy revealed nothing abnormal as to the heart or lungs, and the patient evidently died from syncope in first stage of anæsthesia.

December 17, 1889. Was called to assist in a case of trachelorrhaphy and perineorrhaphy. Mrs. R——, white, aged 37 years. Appears to be in good condition for the operation. Has taken chloroform twice recently for removal of urethral caruncle. When placed upon the operating table the patient is very nervous, and has great fear of the operation. After being reassured as to her danger and having her nerves somewhat calmed, Dr. T——, a surgeon of ability and experience, began the administration of chloroform through an Esmarch's inhaler.

Patient at first took the anæsthetic kindly, but a minute later the face became cyanotic and the respiration labored. Dr. T—— removed the inhaler and felt for the pulse, but found none, and within one minute more all was over. Of course proper measures were taken to revive the patient, but in this, as in the former case, the struggle was against a dead heart. Post mortem was held but revealed nothing of interest.

Two deaths from chloroform in private practice, in one little city within four months! and yet I do not believe blame should be attached to any one connected with these unfortnnate cases. The surgeons in attendance upon them give chloroform every day, and have given it many thousands of times, and these are the first accidents from an anæsthetic they ever witnessed. Let no one suppose that because he has heretofore been fortunate he will necessarily continue to be so; the limitations of our knowledge are the weak places in our pathway, into which the most astute are at any time liable to fall. But misfortune may even be of benefit, for it often promotes diligence and care while the tendency of success is to routine and carelessness.

The fact that a majority of the accidents from anæsthesia have occurred in private practice, naturally leads one to suppose that the general practitioner has from long and successful use of a potent and dangerous agent, become careless as to his methods of administration. This tendency to lack of care and attention to details must be ever watched and guarded against. In the cases related above, no doubt death resulted from cardiac paralysis or "reflex syncope" in the excitement stage of anæsthesia.

This condition of "reflex syncope" is no doubt mainly due to shock produced by fear, and to prove that chloroform may play but a minor part in the fatal drama, the British Medical Journal of October 5, 1889, relates the case of a lady who died after a few inhalations of eau-de-cologne, when she thought she was taking chloroform. In the first case here the fatal issue was partially due to the devitalized condition of the patient, and in the second case I believe it was in part owing to shock induced by fear of the operation. If the second case had any organic heart disease her attending physician failed to detect it, and upon first thought one is inclined to suppose that because the woman had recently taken chloroform twice with no outward symptoms, the deadly dose must have been due to carelessness on the part of the administrator; but we are told by Professor Reeve that "previous inhalations give no guarantee of safety; in a considerable number of the fatal cases there had been previous administrations."

From the most careful study I have been able to make of the subject of general anæsthesia, I deduce the following conclusions: 1. In conditions of extreme shock the surgeon should wait for at least partial reaction before administering an anæsthetic. 2. Shock may be so great as to almost kill the patient, even where there has been no recent injury—the fear of the anæsthetic, the operation, etc., is enough in some cases to put the patient so near the border of death that only a few inhalations of the lethal vapor are required to close the scene. 2. Anæsthesia, except with children and accouchment, should be preceded by a hypodermic injection of morphinæ sulphas 1-6 grain and atropiæ sulphas 1-100 grain. This diminishes shock, strengthens the heart, and promotes and prolongs anæsthesia. 4. Anæsthesia, if undertaken at all, should be complete—the shock of pain and the depressing influence of the emotions may operate fatally in conditions of partial anæsthesia. 5. No anæsthetic is entirely safe, and there is no data from which we can determine with certainty which is the safest anæsthetic—the surgeon generally preferring that one that he has used or seen used most. A few years ago chloroform was somewhat under the ban, but lately it has been gaining ground, and in this country it is the anæsthetic most used in the South and West, while ether is usually preferred in the North and East. 6. Any condition demanding painful surgical interference, is safer with than without anæsthesia, and this is true of subjects suffering with cardiac or pulmonary disease. 7. Anæsthesia is always attended with enough danger to preclude levity and unnecessary remarks at the bedside of the patient; kindness, firmness and sobriety should always distinguish the surgeon in the operating room. 7. Death generally occurs during the first stage of anæsthesia, therefore, the surgeon should be careful not to *drench* the patient, but to establish tolerance, and bring him so gradually under the influence of the anæsthetic that unfavorable symptoms may be noted in their incipiency. 8. Death is usually due to paralysis of the circulatory or respiratory nerve centres; the old idea that chloroform always paralyses the heart and ether the lungs, is not borne out by clinical experience. 9. There are many methods of administering anæsthetics, but that is the best method which secures the patient the freest supply of air, and this is a matter of such vital importance that the surgeon cannot affort to risk a doubt upon it. Right here I will say that a novel and successful method is that of Dr. Vilas, of this city, a surgeon of skill and experience. After putting vasoline on the patient's nose and lips, he places over the face of the patient an ordinary cambric handkerchief, and then drops the chloroform on the tip of the nose, one drop at a time, slowly but steadily, until the anæsthesia is complete. This method secures a sufficiency of air, and with it I have seen Dr. Vilas perform capital operations, without using more than a dram of chloroform. 10. To resuscitate a patient, there should be complete inversion of the body, and this failing, artificial respiration should be resorted to, but nothing will do good when once the heart has become paralysed. 11. With all the care possible, occasionally death will ensue while the patient is on the operating table. Such accidents occurred before ether and chloroform were known, but this should not discourage the surgeon, for after all, artificial anæsthesia remains the greatest blessing that the science of medicine has ever conferred upon mankind.

In the preparation of the foregoing summary I am indebted to the following works: System of Surgery; Gross, Text-book of Surgery; Wyeth, Am. System of Gynecology; Mann, Am. System of Obstetrics; Hirst, Operative Surgery; Stephen Smith, International Encyclopedia of Surgery; Ashhurst, Ref. Handbook of the Medical Sciences; Albert H. Buck, Artificial Anæsthesia and Anæsthetics; Lyman, Medical Record, New York; British Medical Journal; Gaillard's Medical Journal, etc.

